# Educational inequalities in the impact of chronic diseases on exit from paid employment among older workers: a 7-year prospective study in the Netherlands

**DOI:** 10.1136/oemed-2019-105788

**Published:** 2019-08-13

**Authors:** Karen Oude Hengel, Suzan J W Robroek, Iris Eekhout, Allard J van der Beek, Alex Burdorf

**Affiliations:** 1 Erasmus MC Department of Public Health, Rotterdam, The Netherlands; 2 Work, Health & Technology, Netherlands Organisation of Applied Scientific Research TNO, Leiden, The Netherlands; 3 Department of Public and Occupational Health, Amsterdam UMC, VU University Amsterdam, Amsterdam, The Netherlands

**Keywords:** chronic disease, educational inequalities, employment, disability benefits

## Abstract

**Objectives:**

The study aimed to investigate the relative and absolute risks of early exit from paid employment among older workers with a chronic disease, and to assess whether these risks differ across educational groups.

**Methods:**

Data on chronic diseases and demographics from 9160 Dutch workers aged 45–64 years were enriched with monthly information on employment status from Statistics Netherlands. Subdistribution hazard ratios (SHR) and 7-year probabilities among workers with a chronic disease of exit from paid employment through disability benefits, unemployment benefits, early retirement benefits or economic inactivity were estimated using competing risks regression analyses based on Fine and Gray’s models.

**Results:**

Workers with one chronic disease had a higher risk to exit paid employment through disability benefits (SHR 4.48 (95%CI 3.22 to 6.25)) compared with workers without chronic disease, and this risk further increased for multiple chronic diseases (SHR 8.91 (95%CI 6.33 to 12.55)). As occurrence of chronic diseases was highest among low educated workers, the 7-year probabilities to exit paid employment through disability benefits were highest among this group. Cardiovascular, musculoskeletal, psychological and respiratory diseases were associated with disability benefits (SHRs ranging from 2.11 (95%CI 1.45 to 3.07) to 3.26 (95%CI 2.08 to 5.12)), whereas psychological diseases were also related to unemployment (SHR 1.78 (95%CI 1.33 to 2.38)).

**Conclusions:**

Older workers with a chronic disease have a higher risk to exit paid employment through disability benefits. As multimorbidity has an additive effect, addressing multimorbidity as a risk factor for sustainable employment is needed.

**Key messages**

**What is already known about this subject?**

- Workers with a chronic disease are at a higher risk to exit paid employment through disability benefits but not through early retirement. Previous studies found mixed findings for unemployment benefits.
- Workers with a lower educational level are more likely to exit paid employment early than workers with a higher educational level.

**What are the new findings?**

- The occurrence of multimorbidity is high; almost one-third of the workers with a chronic disease reported also another chronic disease.
- The risk for older workers with multiple chronic diseases to exit paid employment through disability benefits was twice as high compared with older workers with one chronic disease, but no significant higher risks were found for any of the other exit routes.
- Cardiovascular, musculoskeletal, psychological and respiratory diseases were significantly related to exit from paid employment through disability benefits. Psychological diseases were also related to unemployment.
- Absolute probabilities to exit paid employment through disability benefits were higher among less educated workers, primarily due to a higher prevalence of chronic diseases among less educated workers.

**How might this impact on policy or clinical practice in the foreseeable future?**

- As multimorbidity is expected to increase due to an ageing working population, interventions are needed to ensure that older workers with multiple chronic diseases are able to remain in the workforce.
- Absolute probabilities, in addition to relative ratios, emphasise the societal need to develop interventions and policies for low educated workers with chronic diseases.

## Introduction

As the population will continue to age in the upcoming decades,^1^ the social and economic realities of an ageing society call for policies aiming at higher and prolonged labour force participation among all individuals of working age.^2^ In recent years, several European countries have implemented measures including discouraging early retirement and raising the statutory retirement age, which requires working until one is older.^3^ Since older workers are more likely to face health problems, the prevalence of individuals within the working age with a chronic disease is expected to grow. In Europe, a substantial part (37%) of the population aged 45–64 years currently live with a long-standing illness or health problem.^4^ Main causes of such health problems are musculoskeletal disorders, cardiovascular diseases, respiratory diseases and mental problems.^5^

People with chronic diseases are less likely to be involved in paid work^6^ for three main reasons: (1) they are not able to start their career, (2) they exit paid employment earlier or (3) they face more difficulties to re-enter paid employment. In Europe, 70% of the individuals with one chronic disease and only 52% of those having multiple chronic diseases work compared with 74% of people without a chronic disease.^7^ Workers with a chronic disease, compared with those without a chronic disease, are at higher risk to exit paid employment through disability benefits^8–11^ but not through early retirement benefits,^8 10 11^ while mixed findings were found for unemployment benefits^8–13^ Almost all studies have investigated the presence of a chronic disease, whereas comorbidity due to multiple chronic diseases has barely been addressed. Evidence for the risk of specific chronic diseases to exit paid employment early through the different pathways is also scarce, with exceptions for musculoskeletal and psychological diseases, which have been shown to be a risk factor for disability and unemployment benefits.^8^

The risk for workers with a chronic disease to exit paid employment through particular pathways may not be similar across educational groups. Previous studies have shown that workers with a low socioeconomic status are more likely to exit paid employment through either disability^14–17^ or unemployment benefits.^17^ These educational differences in disability benefits and unemployment can be explained for 40% and 9%, respectively, by differences in health.^17^

Despite good evidence regarding the link between chronic diseases and early exit from paid employment, through different pathways, this study contributes to the existing literature in several ways. First, it provides additional insights into the effects of chronic diseases on leaving paid employment through different pathways by investigating the differences in having one or multiple chronic diseases and by investigating the effect of specific chronic diseases. Second, this study evaluates whether educational differences in early exit from paid employment among workers with chronic diseases are due to higher prevalence of chronic diseases among low educated workers or to a higher risk to exit paid employment, given the presence of chronic disease. Thereby, the competing risk model of Fine and Gray is applied to provide the subhazard ratios at population level as well as the absolute probabilities at individual level.^18^ In this model, the subhazard ratios for exit from paid employment indicate the ratio of the hazard among workers with one or multiple chronic diseases (ie, exposed group) over the hazard among those without a chronic disease (ie, unexposed group). The model allows direct estimation of the absolute probabilities, which indicate the likelihood, expressed in percentages, to experience an event (ie, to exit paid employment through a specific pathway) regarding specific characteristics of an individual (eg, male workers, married, between 45 and 54 years with multiple chronic diseases). Providing both hazard ratios and absolute probabilities is of importance as it gives additional insight into the possible societal impact that future policies and interventions might have on different target groups.

The objectives of this study were (1) to evaluate the risk of chronic diseases on exit from paid employment due to disability benefits, unemployment benefits, early retirement benefits or economic inactivity, (2) to estimate the 7-year absolute probabilities of these pathways of exit from paid employment and (3) to assess whether these relative risks and 7-year absolute probabilities differ across educational groups and gender.

## Methods

### Data

This study was embedded in the Study on Transitions in Employment, Ability and Motivation (STREAM), which is an Dutch longitudinal study from 2010 onwards. Participants aged 45–64 years annually filled in an online questionnaire on a variety of topics, including sociodemographic factors, work characteristics and health. The study population of STREAM has been extensively described elsewhere.^19^ The Medical Ethical Committee of the VU University Medical Center Amsterdam declared that the Medical Research Involving Human Subjects Act does not apply to STREAM. STREAM data were enriched by Statistics Netherlands with information on main income components, social benefit pensions and gross wages derived from the Dutch tax registers and stored in the social statistical database (SSB).^20^ For the current study, STREAM data of 2010 were matched with monthly information from SSB during 85 months of follow-up (November 2010–December 2017).

### Study population

**Figure 1:**
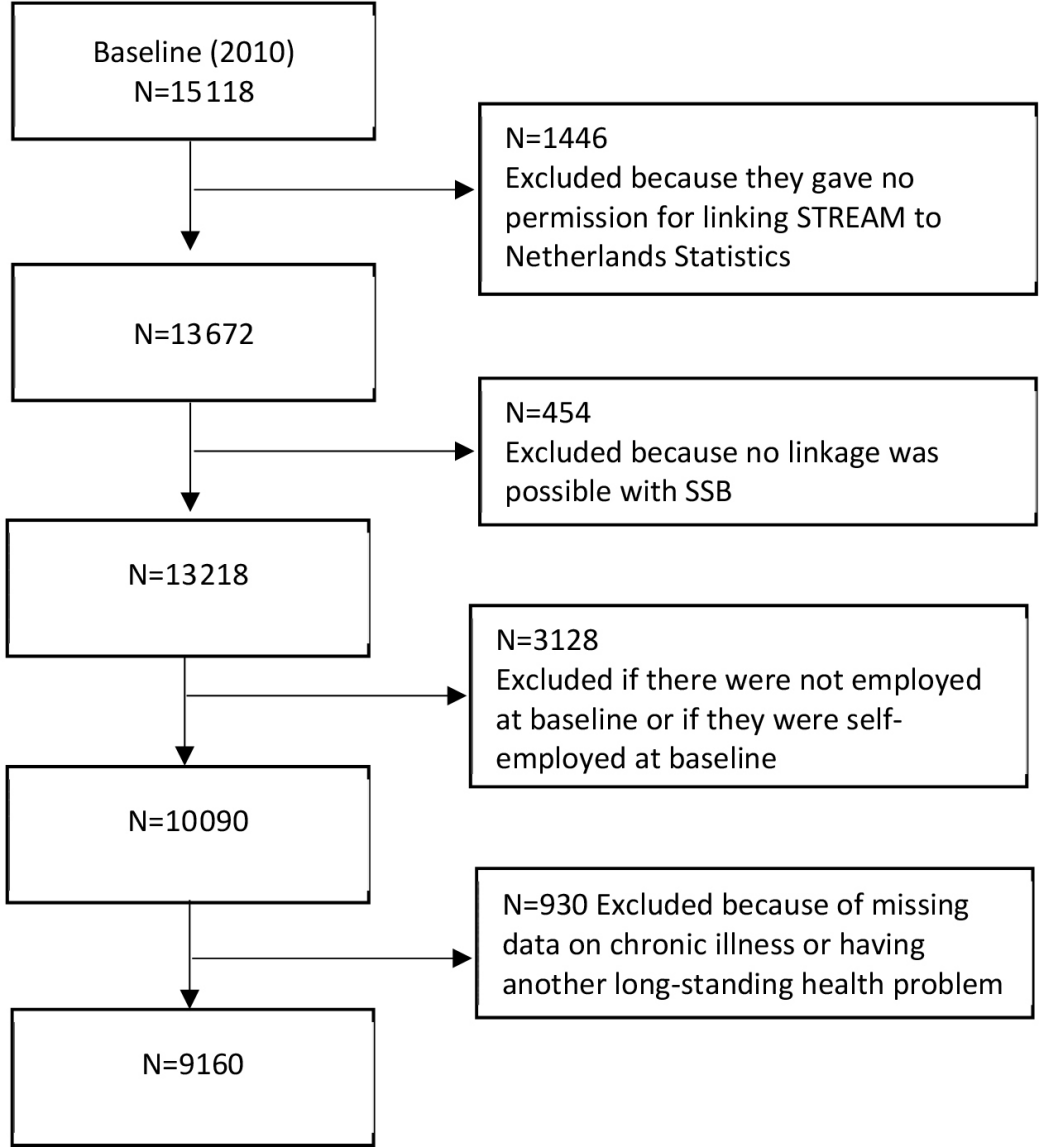
Selection of study population at baseline.

Of the 15 118 STREAM respondents participating in the first wave of STREAM (2010), 13 672 participants gave informed consent to enrich their information with register data of Statistics Netherlands. A small proportion of STREAM respondents (n=454) could not be linked to the registers, either because they could not be identified in the municipal registers or because they did not have a social security number. Individuals were excluded from the analyses if they were not employed or if they were in self-employment. Those with a long-standing health problem other than the six chronic diseases included in the current study (ie, diabetes mellitus, and cardiovascular, digestive, psychological, musculoskeletal and respiratory diseases) were excluded (n=930). The selection resulted in a study population of 9160 persons (figure 1).

### Exit from paid employment

Information on the income components was derived from the Dutch tax register as provided by Statistics Netherlands. Employment status was divided in five mutually exclusive categories: paid employment, disability benefits, unemployment benefits, early retirement benefits and economically inactive. Employed persons had their main income through paid employment. Persons with a disability benefit received a disability benefits for at least 50% of their income. Unemployed persons received unemployment benefits due to losing their job or social security benefits. Early retired persons received a company pension as their main source of income, but had not reached the Dutch statutory retirement age yet. Economically inactive persons did not have personal income or benefits because they stopped for reasons like being a homemaker or retired without receiving early retirement benefits. An individual needed to be in a specific exit pathway for ≥3 months to be included as an actual event for this study. When a participant reported multiple events over time, only the first event in time was considered in the study.

### Chronic diseases

The presence of a chronic disease at baseline was assessed using the following question, ‘Do you (currently) have one or more of the following chronic diseases, disorders or handicaps?’.^21^ Fifteen answer options (ie, chronic diseases, disorders or handicaps) were provided, for which participants could indicate whether these were present. Six chronic diseases were defined by their prevalence and consequences for sickness absence, as described in previous studies^5 10 22^: diabetes mellitus, and cardiovascular, digestive, psychological, musculoskeletal and respiratory diseases. Musculoskeletal diseases were based on three answer options (complaints or disorders (eg, arthritis, rheumatism and repetitive strain injury (RSI)) on (1) hands or arms, (2) legs or feet, or (3) back or neck. If participants answered positively on one or more of these options, they were indicated as have a musculoskeletal disease. Subjects with migraine, skin problems, hearing problems, eye problems, epilepsy and life-threatening diseases were excluded from analysis in the current study. Based on the six included chronic diseases, chronic diseases were categorised into no, one or multiple chronic diseases.

### Sociodemographic variables

Individual characteristics included age, gender, marital status and educational level. Age was categorised into four groups: aged 45–49 years, 50–54 years, 55–59 years and 60–64 years. Marital status was categorised into those living together with a spouse or partner in the same household and others. According to the 1997 International Standard Classification of Education (ISCED),^23^ the highest level of education was categorised into low (primary school, lower secondary school or lower vocational training (ISCED 0–2)), intermediate (intermediate and higher secondary school, or intermediate vocational training (ISCED 3–4)), and high (higher vocational training or university education (ISCED 5–6)).

### Statistical analyses

Baseline statistics were calculated, and differences between low, intermediate and high educated were checked using χ^2^ tests.

The effect of chronic diseases on exit from paid employment during follow-up was analysed using competing risks regression analyses based on Fine and Gray’s proportional subhazard models.^18^ Briefly, the Fine and Gray’s method calculates the cumulative incidence of the likelihood of each pathway and reports subdistribution hazard ratios (SHR) associated with the likelihood to exit paid employment through this pathway while controlling for covariates (ie, age, gender, marital status and educational level) and accounting for other competing events (alternative pathways). An individual was censored at the moment the individual reached the age of 65 years, was missing in SSB during follow-up or at the end of the follow-up period. A SHR-value >1 indicates an increased likelihood of exit from paid employment. Second, interactions between chronic diseases and education on the likelihood to exit paid employment were evaluated in the multivariate survival models by adding the interaction term chronic disease*education to the multivariate model. Because of differences between men and women in the prevalence and type of chronic diseases,^24^ interactions between chronic diseases and gender on the likelihood to exit paid employment were also evaluated. In addition, analyses were performed for the six specific chronic diseases controlling for all covariates in one multivariate model.

Based on the natural logarithm of the SHR and the baseline cumulative subdistribution hazard at 7 years (based on male workers, unmarried, 45–49 years and without chronic diseases), individual risk prediction was calculated in terms of 7-year probabilities for all pathways. This probability was expressed as the percentage to exit paid employment through a specific pathway regarding specific characteristics (eg, having one or multiple chronic diseases, age group, gender and marital status). Differences in absolute probabilities were calculated by subtracting the probabilities of workers without a chronic disease from those with one and multiple chronic diseases.

Analyses were conducted using IBM Statistics IBM SPSS Statistics 25.0 and R-Studio V.1.1.419.

## Results

The study population consisted of 9160 workers, of which a small majority was male (57.3%), the mean age at baseline was 53.8 (SD 5.3) years and more than a quarter had a low education level (26.3%). A chronic disease was significantly more prevalent among low educated workers (58.5%) than among intermediate (56.0%) and high educated workers (50.1%). Among the 54.6% of workers who reported at least one chronic disease, 29.0% also had another chronic disease (29.9% for low educated workers, 29.1% for intermediate workers and 28.0% for high educated workers). Higher prevalence of diabetes, cardiovascular and musculoskeletal diseases was found among lower educated workers than among higher educated workers. In addition, 37.9% of the total study population left paid employment early during the 7 years of follow-up. Exit from paid employment was more prevalent among workers with a low educational level, because disability benefits, unemployment benefits and economic inactivity were more prevalent than in higher educational groups (table 1).

**Table 1:** Characteristics of study population (n=9160) and exit from paid employment through different pathways per educational level

	Educational level					
	Low		Intermediate		High	
	(N=2412)		(N=3531)		(N=3217)	
	n	%	n	%	n	%
Gender						
Male	1317	54.6*	1965	55.6*	1967	61.1*
Age						
45–49	574	23.8*	977	27.7*	841	26.1*
50–54	560	23.2*	1011	28.6*	825	25.6*
55–59	826	34.2*	1055	29.9*	988	30.7*
60–64	452	18.7*	488	13.8*	563	17.5*
Married or cohabitating						
Yes	1933	80.1*	2760	78.2*	2478	77.0*
Chronic disease						
No	1001	41.5*	1555	44.0*	1605	49.9*
One	989	41.0*	1400	39.7*	1161	36.1*
Multiple	422	17.5*	576	16.3*	451	14.0*
Specific chronic diseases						
Cardiovascular	272	11.3*	396	11.2*	316	9.8*
Diabetes Mellitus	223	9.2*	275	7.8*	203	6.3*
Digestive	166	6.9	237	6.7	212	6.6
Musculoskeletal	995	41.3*	1303	36.9*	1012	31.5*
Psychological	99	4.1	166	4.7	147	4.6
Respiratory	191	7.9	313	8.9	259	8.1
Labour force exit						
Disability benefits	115	4.8	156	4.4	82	2.5
Unemployment benefits	356	14.8	472	13.4	368	11.4
Early retirement benefits	424	17.6	558	15.8	624	19.4
Economically inactive	114	4.7	125	3.5	74	2.3

*P<0.05, indicating a significant differences between low, intermediate and high educated workers at baseline values.

Workers with one chronic disease (SHR 4.48, 95% CI 3.22 to 6.25) and with multiple chronic diseases (SHR 8.91, 95% CI 6.33 to 12.55) had a higher risk to exit paid employment through disability benefits compared with workers without a chronic disease, but they had no higher risk for any of the other pathways (table 2). Table 2 shows that cardiovascular diseases (SHR 2.13, 95% CI 1.44 to 3.16), musculoskeletal diseases (SHR 2.80, 95% CI 2.09 to 3.74), psychological diseases (SHR 3.26, 95% CI 2.08 to 5.12) and respiratory diseases (SHR 2.11, 95% CI 1.45 to 3.07) elevated the risk to exit paid employment through disability benefits. Workers with psychological diseases also had a higher risk to become unemployed (SHR 1.78, 95% CI 1.33 to 2.38).

**Table 2:** Influence of chronic diseases assessed at baseline on the likelihood of exit from paid employment during a 7-year follow-up among older Dutch workers (n=9160)

	Disability benefits		Unemployment benefits		Early retirement benefits		Economically inactive*	
	%	SHR (95% CI)†	%	SHR (95% CI)	%	SHR (95% CI)	%	SHR (95% CI)
Chronic disease^‡^								
No	**1.1**	**1.00**	12.8	1.00	15.2	1.00	3.1	1.00
One	**4.8**	**4.48 (3.22 to 6.25**)	13.1	1.02 (0.90 to 1.15)	18.6	1.05 (0.95 to 1.18)	4.0	1.19 (0.94 to 1.51)
Multiple	**9.5**	**8.91 (6.33 to 12.55**)	13.8	1.08 (0.92 to 1.27)	21.7	1.03 (0.90 to 1.18)	3.0	0.91 (0.65 to 1.29)
Specific chronic disease^§^								
Cardiovascular	**6.5**	**2.13 (1.44 to 3.16**)	13.2	1.08 (0.85 to 1.36)	23.5	1.03 (0.86 to 1.23)^2^^¶^	2.4	0.73 (0.40 to 1.31)
Diabetes mellitus	5.7	1.21 (0.75 to 1.95)	14.6	1.06 (0.83 to 1.36)	26.2	1.13 (0.94 to 1.35)	2.4	1.03 (0.61 to 1.75)
Digestive	8.3	0.96 (0.55 to 1.66)	12.5	1.04 (0.49 to 1.38)	18.7	0.93 (0.73 to 1.18)	3.4	0.97 (0.56 to 1.71)
Musculoskeletal	**6.8**	**2.80 (2.09 to 3.74**)	12.9	0.97 (0.84 to 1.12)*	19.8	1.09 (0.97 to 1.23)	3.9	1.05 (0.80 to 1.38)
Psychological	**12.4**	**3.26 (2.08 to 5.12**)^¶^	**18.7**	1.78 (1.33 to 2.38)*	13.6	0.69 (0.46 to 1.02)	4.4	1.28 (0.65 to 2.50)
Respiratory	**7.9**	**2.11 (1.45 to 3.07**)	13.2	1.03 (0.81 to 1.32)	16.6	0.93 (0.74 to 1.15)	3.9	1.06 (0.67 to 1.66)

Significant results (p-value <0.05) are presented in bold.

*Interaction on educational level (p<0.05).[Bibr R2]

†SHR with 95% CI.

‡Analyses are adjusted for gender, age, educational level and marital status.

§Analyses are adjusted for gender, age, educational level, marital status and any other chronic disease.

¶interaction on gender (p<0.05).

SHR, subdistribution hazard ratios.

No interaction effects for educational level or gender were found between chronic diseases and the different pathways out of paid employment. The only exception was an interaction effect for educational level between multiple chronic diseases and economic inactivity. However, after stratification, the risk was not significantly higher for low educated workers with multiple chronic diseases (SHR 1.40, 95% CI 0.40 to 2.33) or lower for intermediate (SHR 0.80, 95% CI 0.44 to 1.47) and high educated workers (SHR 0.50, 95% CI 0.21 to 1.19). Regarding specific chronic diseases, the risk to exit paid employment through unemployment benefits was increased for higher educated workers with psychological diseases (SHR 2.86, 95% CI 1.84; 4.44; Supplementary file A), but not for workers with other educational levels. Regarding interaction effects for gender, male workers with psychological diseases had a higher risk to exit paid employment through disability benefits (SHR 6.77, 95% CI 3.96 to 11.57), while this risk was not significant for female workers with these diseases (SHR 1.18, 95% CI 0.48 to 2.93). Female workers with cardiovascular diseases (SHR 1.46, 95% CI 1.03 to 2.06) were at risk for early retirement benefits, while male workers with cardiovascular diseases had no increased risk for early retirement benefits (SHR 0.92, 95% CI 0.75 to 1.14).

**Figure 2:**
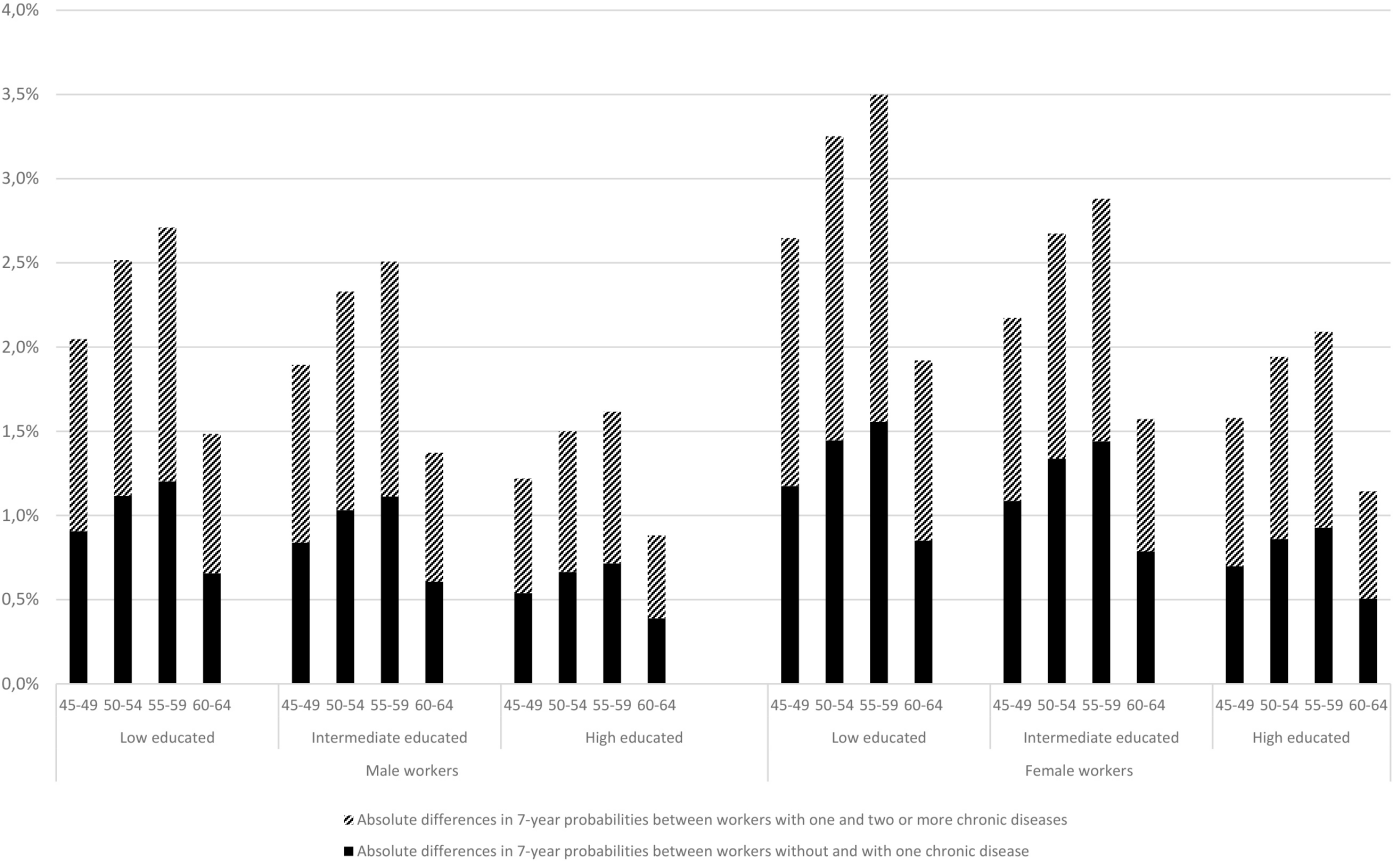
Differences in absolute probabilities to receive disability benefits for male and female without and with one chronic disease (bold) and between one and multiple chronic diseases (striped) presented for each educational level and age group.

Table 3 described the absolute 7-year probabilities to exit paid employment through each specific pathway for workers with no, one or multiple chronic diseases. Irrespective of educational level, the absolute probabilities to exit paid employment through disability benefits among those with one and multiple chronic diseases increased up to 55–59 years, where after the absolute probabilities decreased. The differences in absolute probabilities to receive disability benefits between workers with and without multiple chronic diseases was twice as high compared with these differences between those with and without one chronic disease (figure 2). The (differences in) 7-year probabilities to receive disability benefits for chronic diseases were highest among low educated workers. The differences in 7-year probability to exit paid employment through unemployment benefits, early retirement benefits and economic inactivity between workers without or with one or multiple chronic diseases were very small across all groups.

**Table 3:** 7-year probability (%) of exit from paid employment through different pathways for male and female workers married or cohabitating without no, one and multiple chronic diseases, stratified for educational level and age group

	Disability benefits			Unemployment benefits			Early retirement benefits			Economic inactive		
N of chronic diseases	0	1	≥2	0	1	≥2	0	1	≥2	0	1	≥2
Male												
Low educated												
45–49	0.3	1.2	2.3	8.2	8.3	8.8	0.1	0.1	0.1	1.1	1.4	1.0
50–54	0.3	1.4	2.8	9.7	9.8	10.4	0.9	0.9	0.9	1.3	1.5	1.1
55–59	0.3	1.5	3.1	10.7	10.9	11.5	6.4	6.7	6.6	1.4	1.6	1.2
60–64	0.2	0.8	1.7	5.4	5.5	5.8	31.4	32.8	32.3	1.1	1.3	1.0
Intermediate educated												
45–49	0.2	1.1	2.1	7.2	7.4	7.8	0.1	0.1	0.1	0.9	1.0	0.8
50–54	0.3	1.3	2.6	8.5	8.7	9.2	1.0	1.1	1.0	0.9	1.1	0.9
55–59	0.3	1.4	2.8	9.5	9.7	10.2	7.1	7.5	7.4	1.0	1.2	0.9
60–64	0.2	0.8	1.5	4.7	4.8	5.1	34.5	36.0	35.4	0.8	1.0	0.8
High educated												
45–49	0.2	0.7	1.4	6.3	6.4	6.7	0.1	0.1	0.1	0.6	0.7	0.6
50–54	0.2	0.9	1.7	7.4	7.5	8.0	1.1	1.2	1.2	0.7	0.8	0.6
55–59	0.2	0.9	1.8	8.3	8.4	8.9	7.9	8.3	8.2	0.7	0.9	0.7
60–64	0.1	0.5	1.0	4.1	4.2	4.4	37.6	39.2	38.5	0.6	0.7	0.5
Female												
Low educated												
45–49	0.3	1.5	3.0	8.7	8.9	9.4	0.1	0.1	0.1	3.6	4.2	3.3
50–54	0.4	1.9	3.7	10.3	10.5	11.1	0.8	0.8	0.8	3.9	4.6	3.5
55–59	0.5	2.0	4.0	11.4	11.6	12.3	5.7	6.0	5.9	4.2	5.0	3.9
60–64	0.2	1.1	2.2	5.7	5.8	6.2	28.5	29.8	29.3	3.4	4.1	3.1
Intermediate educated												
45–49	0.3	1.4	2.8	7.7	7.8	8.3	0.1	0.1	0.1	2.7	3.2	2.4
50–54	0.4	1.7	3.4	9.1	9.3	9.8	0.9	0.9	0.9	2.9	3.5	2.7
55–59	0.4	1.9	3.7	10.1	10.3	10.9	6.4	6.7	6.6	3.2	3.8	2.9
60–64	0.2	1.0	2.0	5.1	5.2	5.4	31.4	32.8	32.2	2.6	3.1	2.4
High educated												
45–49	0.2	0.9	1.8	6.7	6.8	7.2	0.1	0.1	0.1	1.9	2.3	1.7
50–54	0.2	1.1	2.2	7.9	8.0	8.5	1.0	1.1	1.0	2.1	2.5	1.9
55–59	0.3	1.2	2.4	8.8	9.0	9.5	7.1	7.4	7.1	2.3	2.7	2.1
60–64	0.1	0.7	1.3	4.4	4.5	4.7	34.2	35.7	34.2	1.8	2.2	1.7

## Discussion

Workers with one chronic disease had a higher risk to exit paid employment through disability benefits, and this risk further increased for multiple chronic diseases. Workers with chronic diseases showed no elevated risk to exit paid employment through any of the other pathways (unemployment benefits, early retirement benefits and economically inactive). Due to higher prevalence of chronic diseases among low educated workers, the 7-year probabilities to exit paid employment due to disability benefits were highest among low educated workers with multiple chronic diseases.

As expected, and within the range of previous findings,^8 9 11 13^ workers with one chronic disease had a higher risk to exit paid employment through disability benefits. In addition, multimorbidity had an additive effect on leaving paid employment through disability benefits, whereby the risk for workers with multimorbidity to receive disability benefits was almost twice as high compared with those with one chronic disease. A recent study also found that workers with multimorbidity were more prone to exit paid employment early than those with one or no chronic disease.^25^ Workers suffering two or more chronic diseases might become more or earlier functionally impaired, which will impede their working life. A previous study showed that multiple mental and physical chronic diseases indeed increased the risk of work loss.^26^ However, the health consequences of multimorbidity in the working population are still poorly understood as specific combinations of chronic diseases might have a stronger health impact than other combinations.^27^ This study highlights the importance of addressing multimorbidity on leaving paid employment in future research.

Workers with chronic diseases had in general no higher risk to exit paid employment through unemployment benefits, early retirement benefits or economic inactivity. It should be noticed that the economic recession took place during the data collection. This also appeared in higher unemployment rates compared with studies conducted before this period,^12 17 28^ and unemployment is therefore less likely to be health-driven. The results corroborate with a study on unemployment among workers with and without chronic diseases conducted during and after the peaks of recessions in 1986 and 1990.^29^ Regarding early retirement, this pathway is voluntary whereby worker’s decision to retire early also relies more on social and financial factors.^30–32^ Moreover, before the economic incentives to retire early were restricted in 2005, early retirement and disability benefits acted as communicating vessels to exit paid employment early.^33^ Because data of the current study were collected from 2010 onwards, receiving early retirement benefits might be no longer automatically considered as a substitute of disability benefits for workers with chronic diseases.

The highest risks and absolute probabilities to exit paid employment through disability and unemployment benefits were found for workers with psychological diseases. Previous studies showed somewhat different risk estimates but in the same direction.^8 11 34^ These differences in estimates could be explained by differences in the definition, different study populations and different statistical methods. Almost 30% of the workers with psychological diseases in this study left paid employment through disability and unemployment benefits, and workers once receiving disability benefits due to mental diseases are less likely to re-enter paid employment. In addition, the prevalence of musculoskeletal diseases, especially among low educated workers, is highest in the current study. In line with previous findings,^8 11^ workers with musculoskeletal diseases had a higher risk to exit paid employment through disability benefits. Besides, several interactions effects were found between specific chronic diseases and educational level or gender on leaving paid employment. However, as many models were tested for interaction effects, the possibility of finding effects by chance might be an explanation.

By applying the Fine and Gray’s model, this study was able to provide relative ratios at population level and absolute probabilities at individual level. While the relative ratios to exit paid employment through disability benefits did not differ across educational groups, the 7-year probabilities to receive disability benefits were somewhat higher among low educated workers in all age groups. Because of the lower employment levels of low educated workers with chronic diseases,^6^ their higher risk for early exit^14 17^ and their higher prevalence of chronic diseases,^35^ the differences in 7-year probabilities emphasise the need for interventions tailored to this vulnerable group of workers.

Previous studies showed inconsistency as to which factors modify the relation between chronic disease and early exit from paid employment.^10 11^ While these studies showed that different psychological work-related factors modify the risk for a health-related exit, mixed findings were reported for work factors as effect modifier to exit paid employment through unemployment and early retirement. However, as chronic disease may influence work demands as well as leaving paid employment, future research could further investigate the interplay between chronic disease, working conditions, educational level and early exit from paid employment.

This is one of the first studies investigating the effect of chronic diseases on exit from paid employment by presenting both the relative and absolute risks. Presenting absolute probabilities and relative risks is important for decision makers to better estimate the possible effects of interventions and the societal value. Another strength of this longitudinal study is the 7-year follow-up period with the use of reliable monthly register data to determine employment status instead of the yearly self-reported employment status. Some limitations should be noted as well. First, data on chronic disease were self-reported and could therefore be a combination of disorders and complaints as the question did not specify whether the chronic disease was diagnosed by a physician. Moreover, chronic diseases were inquired in STREAM without any further definition or specification of the disease, which could lead to a lower validity regarding the assessment of a chronic disease. Second, this study did not assess the functional limitation that impeded the work activity of workers with a chronic disease. As functional limitations will vary by chronic disease, it is recommended to include functional limitations in future questionnaires as well. Third, it should be acknowledged that the current study population of workers of 45 years and older with a chronic disease was a healthy selection of the total population as they already had managed to continue working until to this age. Lastly, if and when workers returned to paid employment was not considered. Even though workers can always return to work after displacement through any of the pathways, it is likely to be relatively low in a study population of 45 years and older. This hypothesis is in line with a recent Dutch study that showed that almost 90% of workers aged 45–54 years continued to receive disability benefits after 5 years.^36^

To conclude, multimorbidity has an additive effect as workers with multiple chronic disease were almost twice as likely to exit paid employment through disability benefits than workers with one chronic disease. Because the proportion of workers with multiple chronic diseases is expected to increase, policies and interventions are needed to ensure that older workers with multiple chronic disease are able to remain in the workforce. Educational inequalities in exit from paid employment through disability benefits were observed in absolute 7-year probabilities, primarily due to a higher occurrence of chronic diseases among low educated workers, and interventions are therefore needed for this group of vulnerable workers.
